# Supplementary material for: The rodent vaginal microbiome across the estrous cycle and the effect of genital nerve electrical stimulation
Source: PLoS One. 2020 Mar 12;15(3):e0230170. doi: 10.1371/journal.pone.0230170 (PMC7067422; doi:10.1371/journal.pone.0230170)
Supplement: S2 Table — (DOCX) [file pone.0230170.s002.docx]

**S2 Table.**

|  |  | All samples | | Proestrus | | Estrus | | Metestrus | | Diestrus | |
| --- | --- | --- | --- | --- | --- | --- | --- | --- | --- | --- | --- |
|  | Genus | B:T Ratio | *p*-value | B:T Ratio | *p*-value | B:T Ratio | *p*-value | B:T Ratio | *p*-value | B:T Ratio | *p*-value |
| OTU1 | *Proteus* | 0.913 | 0.491 | 1.000 | 1.000 | 0.952 | 0.852 | 0.758 | 0.315 | 0.963 | 0.912 |
| OTU2 | *Escherichia/Shigella* | 1.342 | 0.027 | 2.100 | 0.030 | 1.403 | 0.152 | 1.500 | 0.156 | 0.850 | 0.529 |
| OTU3 | *Streptococcus* | 1.313 | 0.041 | 0.732 | 0.429 | 1.350 | 0.205 | 1.704 | 0.043 | 1.295 | 0.315 |
| OTU4 | *Morganella* | 0.857 | 0.227 | 0.885 | 0.792 | 0.832 | 0.437 | 0.919 | 0.780 | 0.765 | 0.315 |
| OTU5 | *Pasteurellaceae* | 1.119 | 0.396 | 1.848 | 0.082 | 0.801 | 0.347 | 1.344 | 0.278 | 1.132 | 0.631 |
| OTU6 | *Enterococcus* | 1.036 | 0.791 | 1.063 | 0.931 | 0.994 | 0.979 | 0.979 | 0.968 | 1.199 | 0.481 |
| OTU7 | *Corynebacterium* | 1.622 | < 0.001 | 0.805 | 0.537 | 0.577 | 0.016 | 0.488 | 0.008 | 0.641 | 0.089 |
