# Supplementary material for: The rodent vaginal microbiome across the estrous cycle and the effect of genital nerve electrical stimulation
Source: PLoS One. 2020 Mar 12;15(3):e0230170. doi: 10.1371/journal.pone.0230170 (PMC7067422; doi:10.1371/journal.pone.0230170)
Supplement: S3 Table — (DOCX) [file pone.0230170.s003.docx]

**S3 Table.**

|  |  | All samples | | Proestrus | | Estrus | | Metestrus | | Diestrus | |
| --- | --- | --- | --- | --- | --- | --- | --- | --- | --- | --- | --- |
|  | Genus | B:T Ratio | *p*-value | B:T Ratio | *p*-value | B:T Ratio | *p*-value | B:T Ratio | *p*-value | B:T Ratio | *p*-value |
| OTU1 | *Proteus* | 1.066 | 0.560 | 1.375 | 0.371 | 1.308 | 0.241 | 0.772 | 0.368 | 1.068 | 0.699 |
| OTU2 | *Escherichia/Shigella* | 0.997 | 0.976 | 1.214 | 0.594 | 0.703 | 0.100 | 0.788 | 0.407 | 1.151 | 0.413 |
| OTU3 | *Streptococcus* | 1.236 | 0.056 | 1.058 | 0.859 | 1.695 | 0.020 | 1.123 | 0.680 | 0.920 | 0.624 |
| OTU4 | *Morganella* | 0.813 | 0.057 | 1.318 | 0.440 | 0.914 | 0.698 | 0.725 | 0.267 | 0.650 | 0.011 |
| OTU5 | *Pasteurellaceae* | 1.027 | 0.799 | 0.963 | 0.953 | 0.854 | 0.478 | 1.152 | 0.581 | 1.198 | 0.273 |
| OTU6 | *Enterococcus* | 0.831 | 0.090 | 0.318 | 0.440 | 0.905 | 0.664 | 0.756 | 0.332 | 0.799 | 0.187 |
| OTU7 | *Corynebacterium* | 1.187 | 0.049 | 0.517 | 0.019 | 0.788 | 0.280 | 0.877 | 0.630 | 0.895 | 0.481 |
